# Supplementary material for: In-plane quasi-single-domain BaTiO3 via interfacial symmetry engineering
Source: Nat Commun. 2021 Nov 22;12:6784. doi: 10.1038/s41467-021-26660-7 (PMC8608839; doi:10.1038/s41467-021-26660-7)
Supplement: Supplementary file 1 — Supplementary Information [file 41467_2021_26660_MOESM1_ESM.pdf]

Supplementary Information for

In-plane quasi-single-domain $BaTiO_3$ *via* interfacial symmetry engineering

J. W. Lee[1,9], K. Eom[1,9], T. R. Paudel[2,3], B. Wang[4], H. Lu[2], H. X. Huyan[5], S. Lindemann[1], S. Ryu[1], H. Lee[1], T. H. Kim[1], Y. Yuan[4], J. A. Zorn[4], S. Lei[4], W. P. Gao[5], T. Tybell[6], V. Gopalan[4], X. Q. Pan[5,7,8], A. Gruverman[2], L. Q. Chen[4], E. Y. Tsymbal[2], and C. B. Eom[1*]

[1]Department of Materials Science and Engineering, University of Wisconsin-Madison, Madison, Wisconsin 53706, USA

[2]Department of Physics and Astronomy & Nebraska Center for Materials and Nanoscience, University of Nebraska, Lincoln, Nebraska 68588, USA

[3]Department of Physics, South Dakota School of Mines and Technology, Rapid City, South Dakota 57701, USA

[4]Department of Materials Science and Engineering, The Pennsylvania State University, University Park, Pennsylvania 16802, USA

[5]Department of Materials Science and Engineering, University of California, Irvine, California 92697, USA

[6]Department of Electronic Systems, Norwegian University of Science and Technology, 7491 Trondheim, Norway

[7]Department of Physics and Astronomy, University of California, Irvine, California 92697, USA

[8]Irvine Materials Research Institute, University of California, Irvine, California 92697, USA

[9]These authors contributed equally to this work.

* Corresponding author. Email: eom@engr.wisc.edu

Supplementary Figures

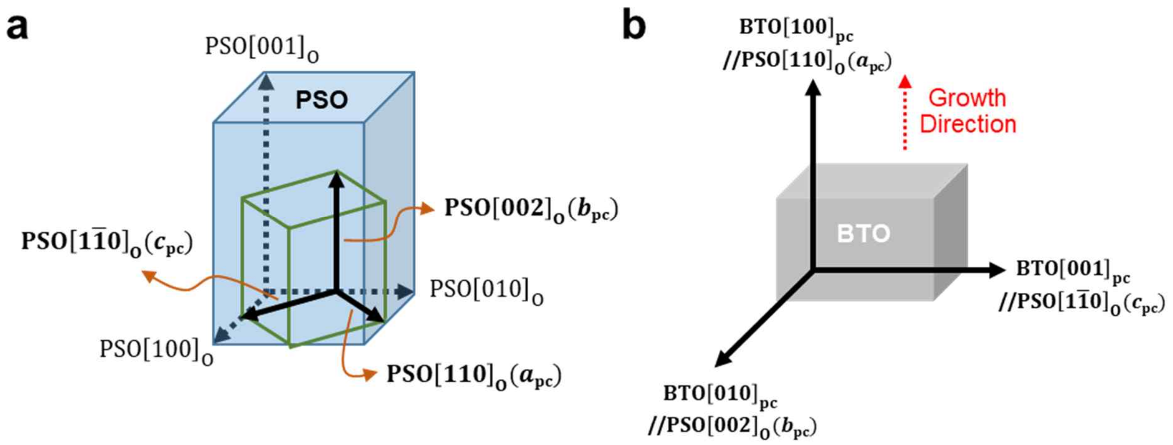

Supplementary Figure 1. Axis notation for BTO and PSO. a, Schematic diagram showing PSO crystal structure in orthorhombic unit cell. The inner cube is the pseudocubic unit cell. **b**, The epitaxial arrangement of $(100)_{pc}$-oriented BTO on PSO $(110)_{O}$ substrate. The growth direction is oriented along the PSO $[110]_{O}$ (or BTO $[100]_{pc}$). Note that the BTO $[001]_{pc}$ and BTO $[100]_{pc}$ directions are not exactly parallel with the c_{pc} (PSO $[1\bar{1}0]_{O}$) and a_{pc} (PSO $[110]_{O}$) directions, respectively, due to the monoclinic nature of the pseudocubic PSO unit cell.

Supplementary Note 1: DFT calculations

DFT calculations were performed with two different initial polarizations of BTO with corresponding two monoclinic tilt directions: the opposite tilt direction (Fig. 2a) and the same tilt direction (Supplementary Figure 2a). The results show that the BTO/PSO with the opposite tilting direction (Fig. 2a) has lower energy than that of the same tilting direction (Supplementary Figure 2a) by 18.6 mJ/m^2. The origin of such a lower energy state is associated to the interfacial Sc-O-Ti network. It should be noted that the bulk PSO has Sc-O-Sc bonding angle of ~147° with ScO_6 octahedral rotation pattern ($a^-a^-c^+$ in Glazer's notation)[1-3], while bulk BTO does not have any rotation/tilt at room temperature[4]. In the BTO/PSO heterostructure, octahedral tilts can propagate into the BTO layers near the interface region[4]. The calculated Sc-O-Ti angles are 171°, 173° in the case of the opposite tilt direction (Supplementary Figure 2b) and 176°, 168° in the case of the same tilt direction (Supplementary Figure 2c), respectively. Relatively large angle difference (~8°) between two adjacent Sc-O-Ti bond angles in the same tilt direction is likely to result in the higher energy state.

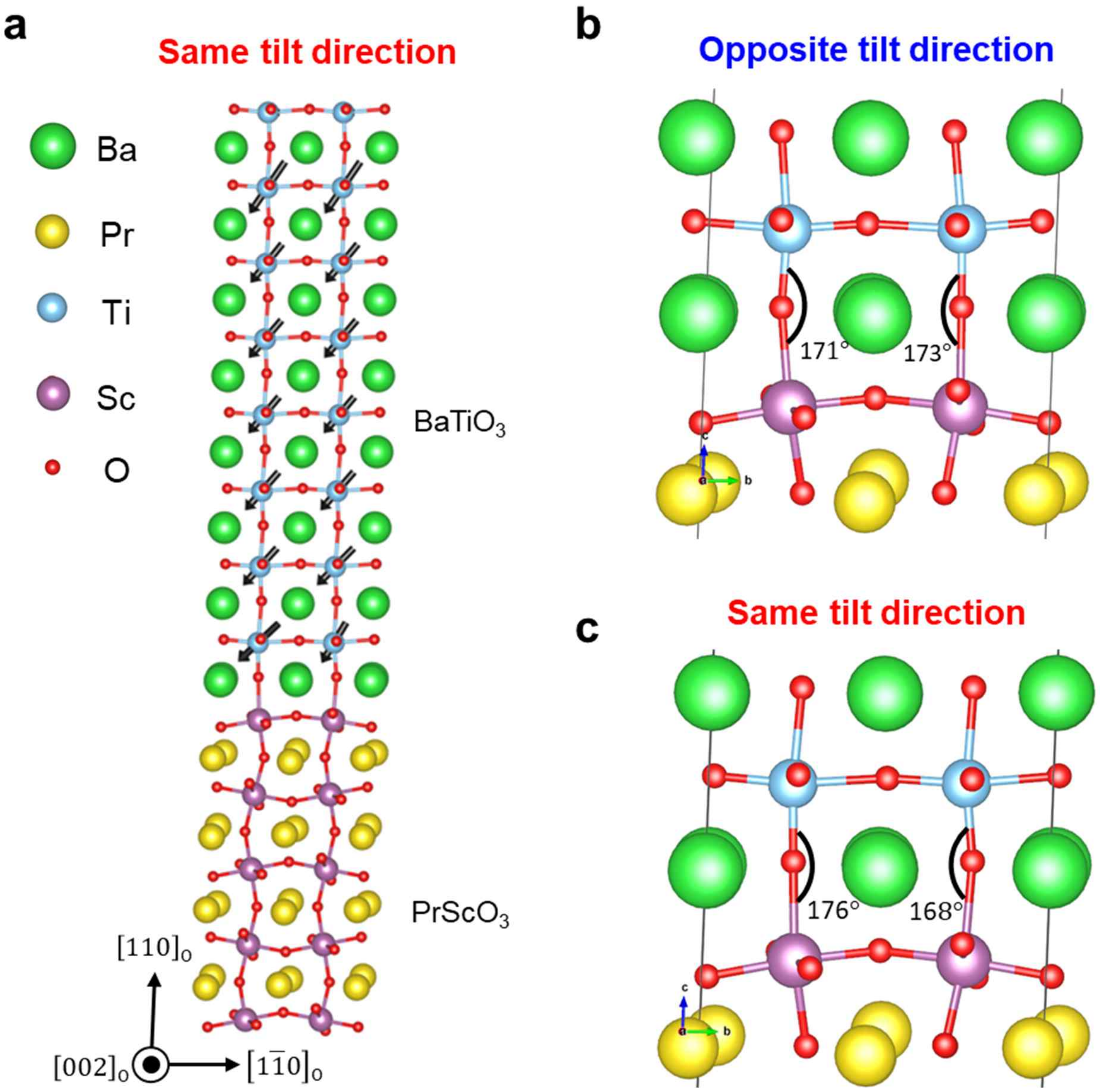

Supplementary Figure 2. Density functional theory calculations for BTO/PSO heterostructure. a, The relaxed atomic structure by DFT calculation in the case of the same tilt direction. The initial Ti-displacement was set to the diagonal direction (i.e., combination of PSO $[\bar{1}\bar{1}0]_O$ and $[\bar{1}10]_O$). **b**, **c**, The interfacial atomic structure with the bond angle between Sc-O-Ti in the case of (**b**) the opposite tilt direction and (**c**) the same tilt direction, respectively.

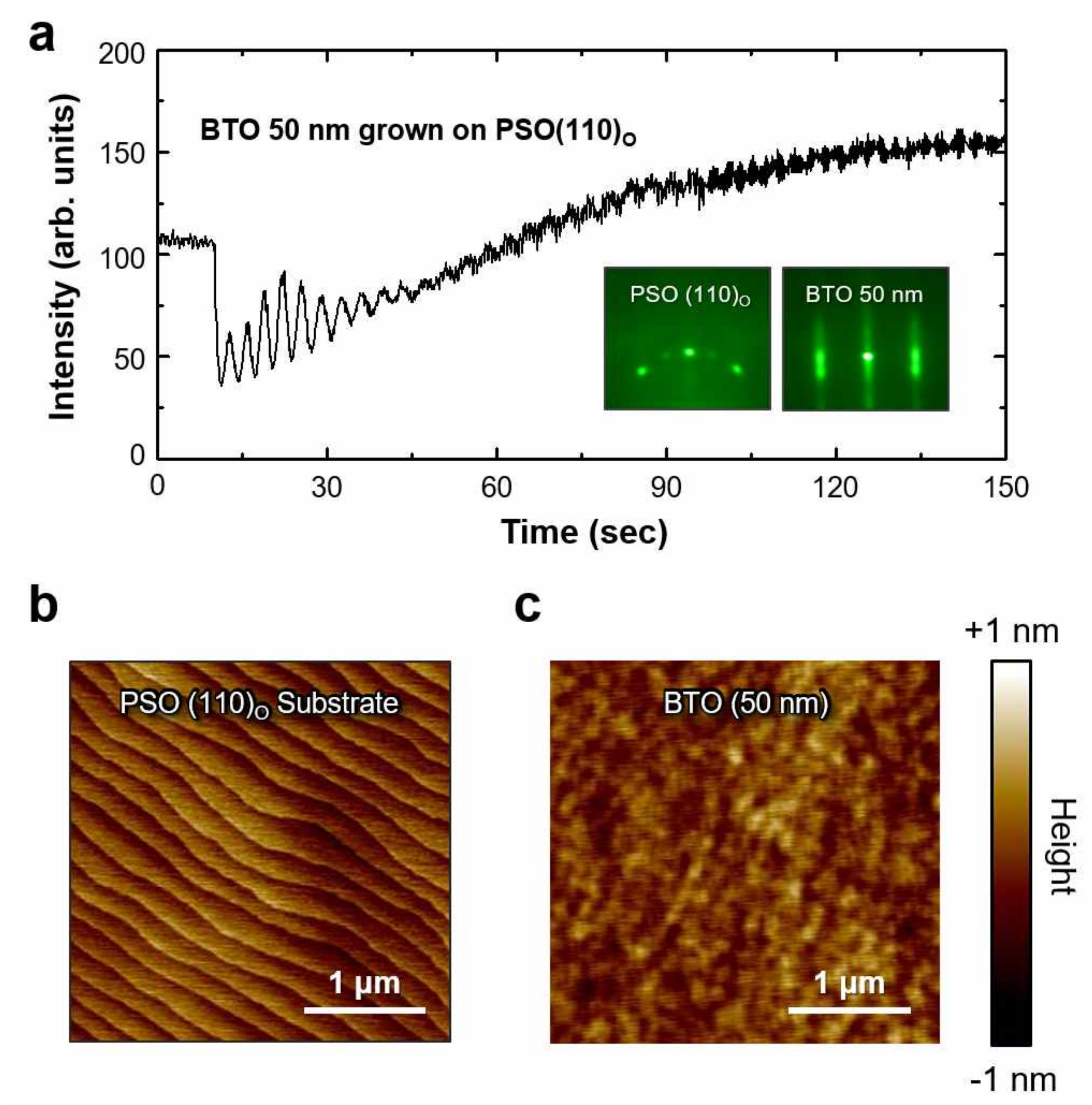

Supplementary Figure 3. In-situ RHEED observation and surface topography. a, RHEED oscillations for the growth of BTO. The data from initial growth (~140 seconds) is represented. The insets show the RHEED patterns of a PSO $(110)_O$ substrate and a 50-nm-thick BTO film. **b**, **c**, AFM topography images of a treated PSO $(110)_O$ substrate (**b**) and 50-nm-thick BTO film on PSO $(110)_O$ substrate (**c**).

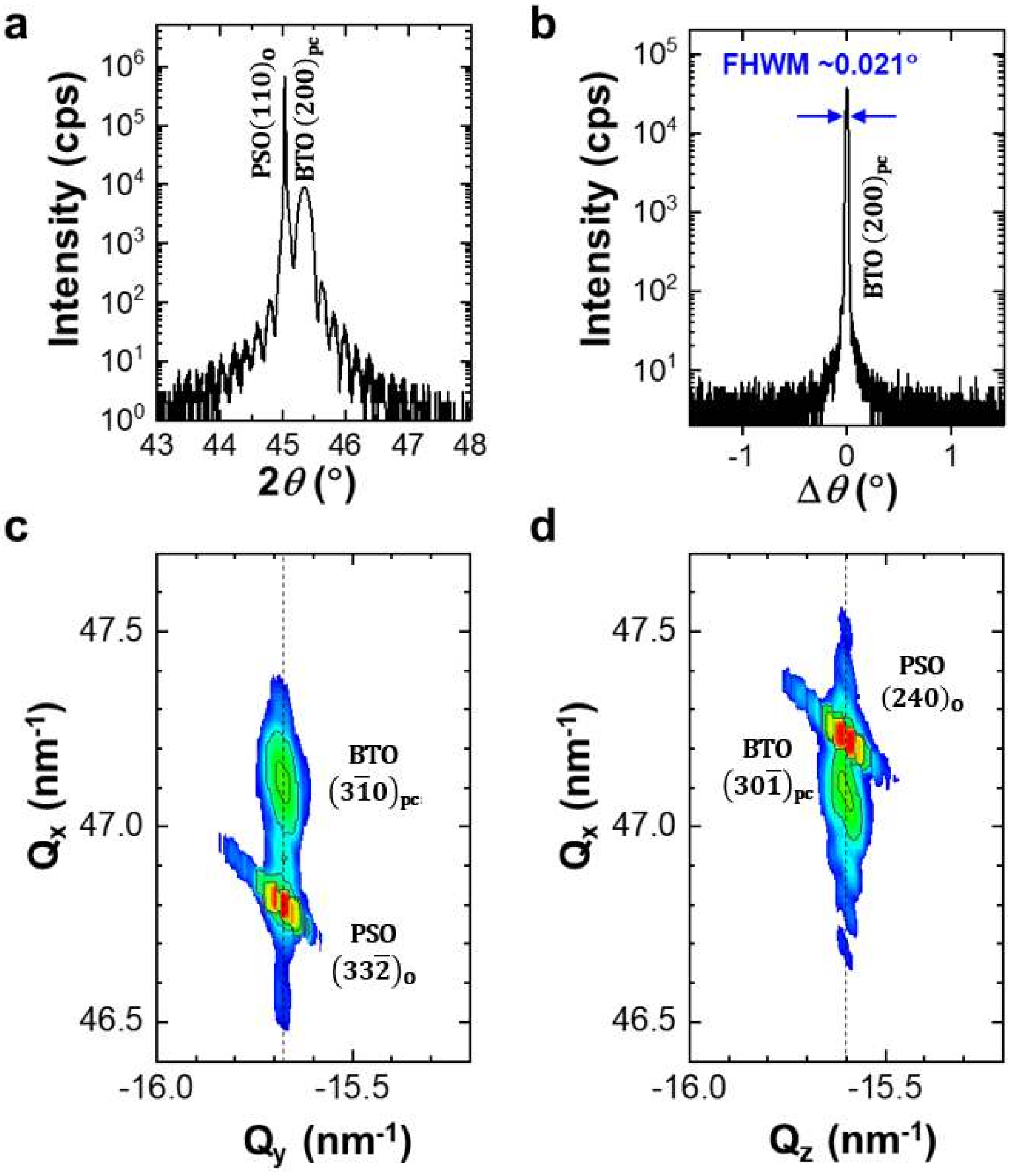

Supplementary Figure 4. X-ray diffraction patterns of epitaxial BTO films grown on PSO $(110)_O$ substrates. a, **b**, (**a**) Out-of-plane θ-2θ measurement, (**b**) a BTO $(200)_{pc}$ rocking curve of the BTO film. **c**, **d**, Reciprocal space maps of the BTO film around PSO $(33\bar{2})_O$ (**c**) and PSO $(240)_O$ (**d**) Bragg peaks, respectively.

Supplementary Note 2: The overall polarization state of the BTO film

In addition to interface and middle region of the BTO film, the polarization state in the top region of the BTO film (close to the surface) is also shown in the Supplementary Figure 5. It is revealed that in-plane polarization direction of BTO film is toward PSO $[1\bar{1}0]_O$ (Supplementary Figure 2c, which is in agreement with the middle region (Fig. 3e) and near interface region (Fig. 3f). The average Ti displacement is estimated to 6.9 $\pm$ 1.3 pm, which is comparable to the values in the middle region of the film (6.0 $\pm$ 1.1 pm) and BTO/PSO interface region (6.0 $\pm$ 1.0 pm) within the error range. This uniform in-plane Ti displacement is mainly attributed to the coherent nature of BTO film on PSO substrate (Supplementary Figure 4c, d). In addition, there is a tiny amount of out-of-plane component pointing upward (toward top surface; 1.9 $\pm$ 1.1 nm). We think that this tiny amount of out-of-plane component pointing upward is presumably originated from external factors, e.g, the electronic boundary condition formed by surface adsorbates such as H_2O[5].

The overall displacement maps near interface, middle and top regions of the BTO film are also shown in Supplementary Figure 6. It is clearly seen that the in-plane polarization direction is along PSO $[1\bar{1}0]_O$ in all images. While there is a dominant downward polarization (out-of-plane polarization toward PSO $[\bar{1}\bar{1}0]_O$ direction) near interface due to interfacial electrostatic potential, the randomly-oriented out-of-plane polarization is observed in the middle region.

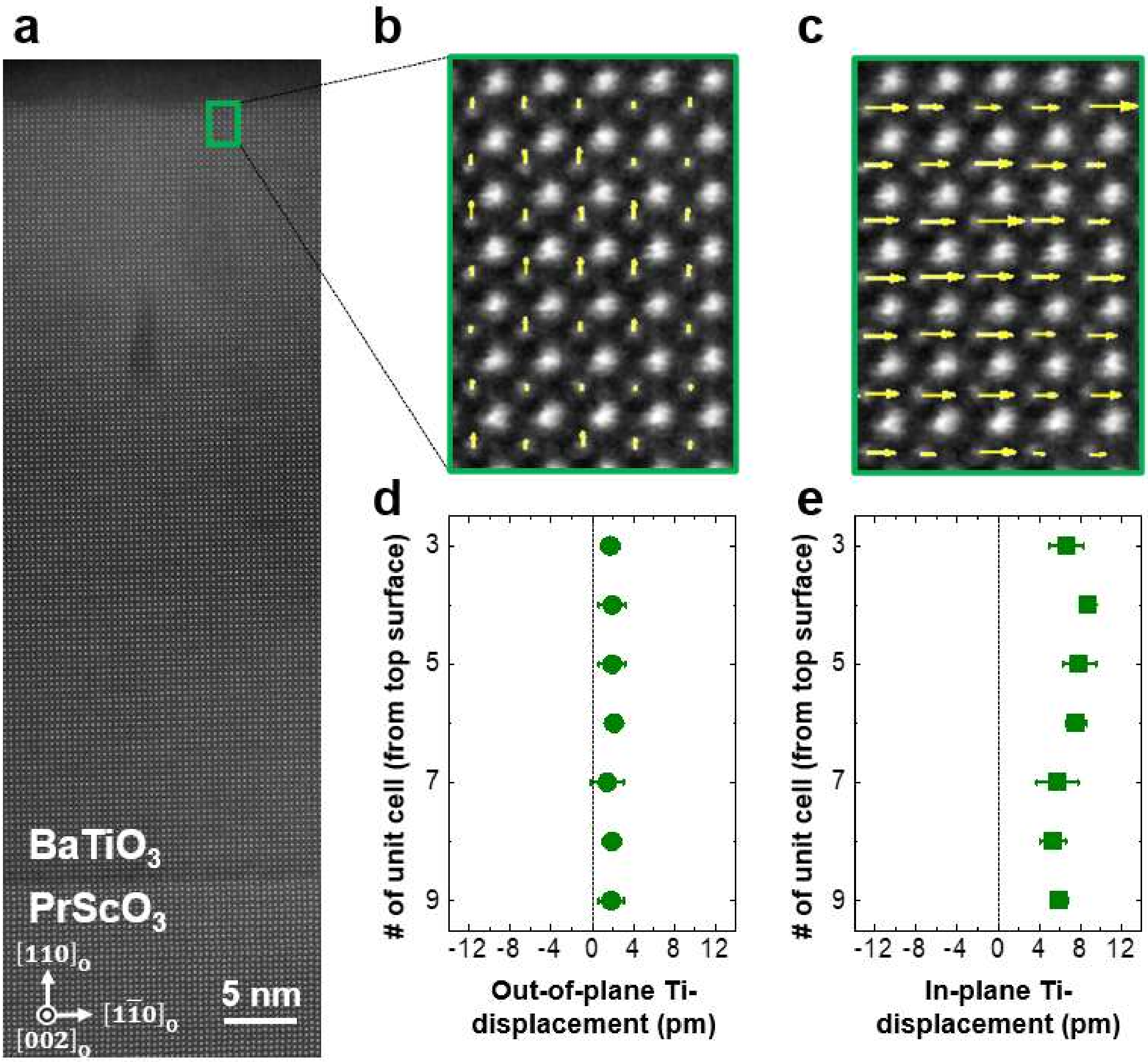

Supplementary Figure 5. STEM measurements of the top region of BTO film on PSO $(110)_O$ substrate. **a**, A low magnification HAADF-STEM image of BTO film with a zone axis of PSO $[002]_O$. **b**, **c**, High resolution images showing (**b**) out-of-plane and (**c**) in-plane components of Ti displacement in the top region of the BTO film, which is marked in green color in (**a**). Note that the size of arrows corresponds to the amount of Ti displacement. **d**, **e**, Ti displacement of (**d**) in-plane and (**e**) out-of-plane component as a function of BTO unit cell from the top surface.

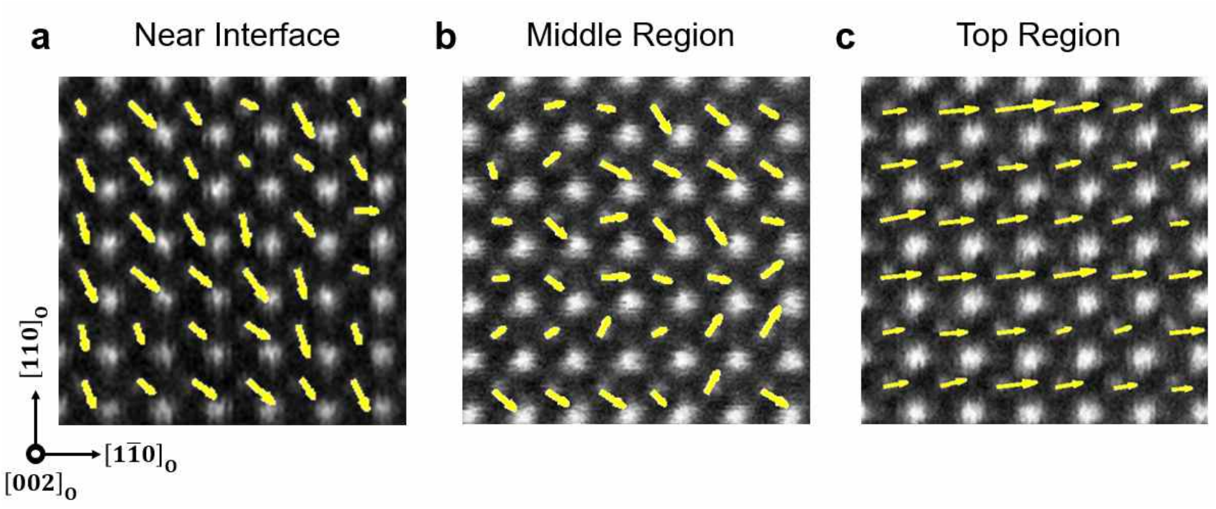

Supplementary Figure 6. Overall polarization state of the BTO film on PSO $(110)_O$ substrate. a-c, A high resolution HAADF-STEM images with a zone axis of PSO $[002]_O$ showing overall polarization low magnification HAADF-STEM image of BTO film at (**a**) the interface region, (**b**) the middle of the film, and (**c**) the top region of the film, respectively.

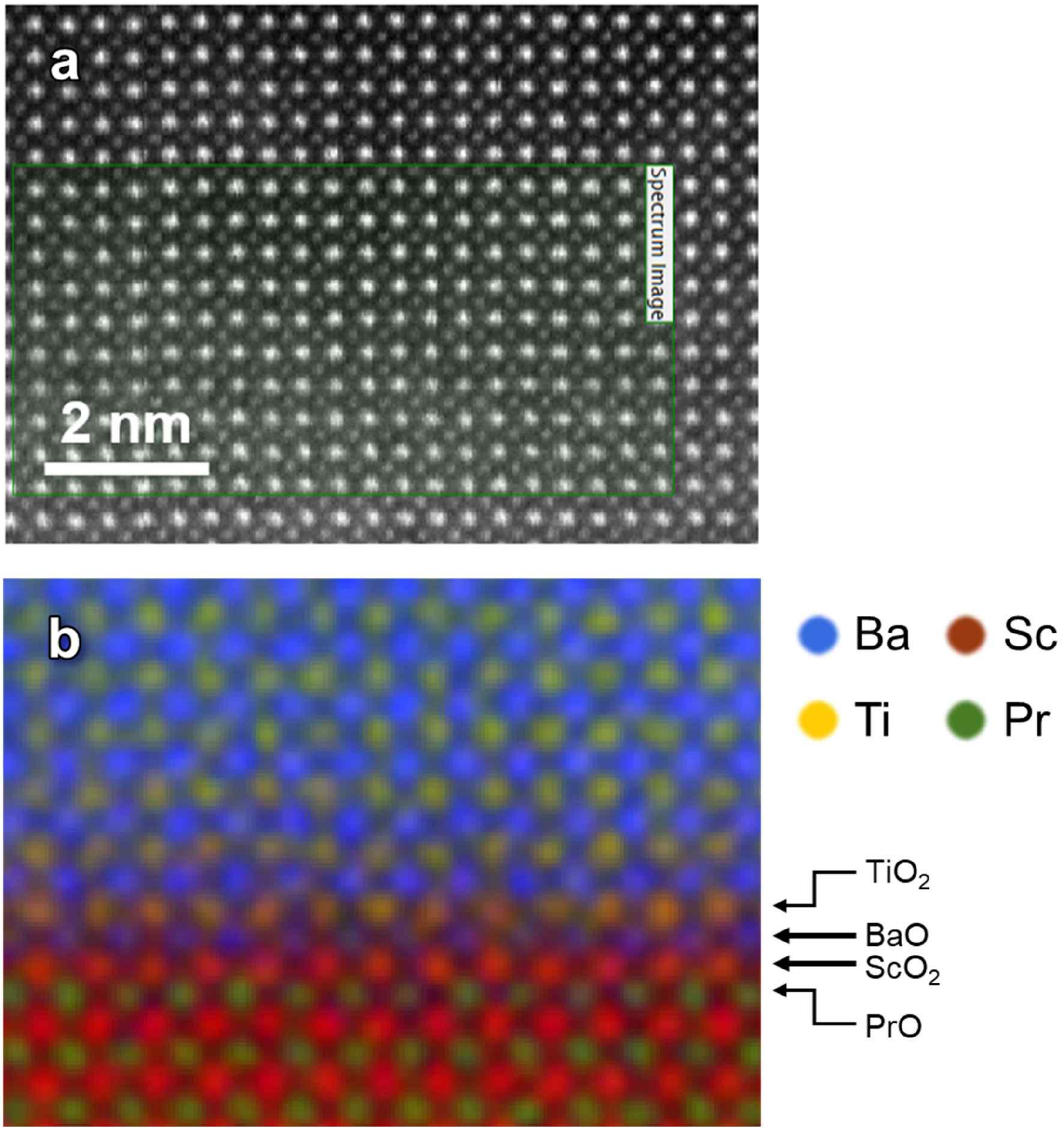

Supplementary Figure 7. Compositional analysis near the interface region between BTO and PSO. **a**, HAADF-STEM images with a zone axis of PSO $[002]_O$. **b**, Energy dispersive spectroscopy (EDS) elemental mapping images indicating ScO_2-termination of PSO $(110)_O$ substrate.

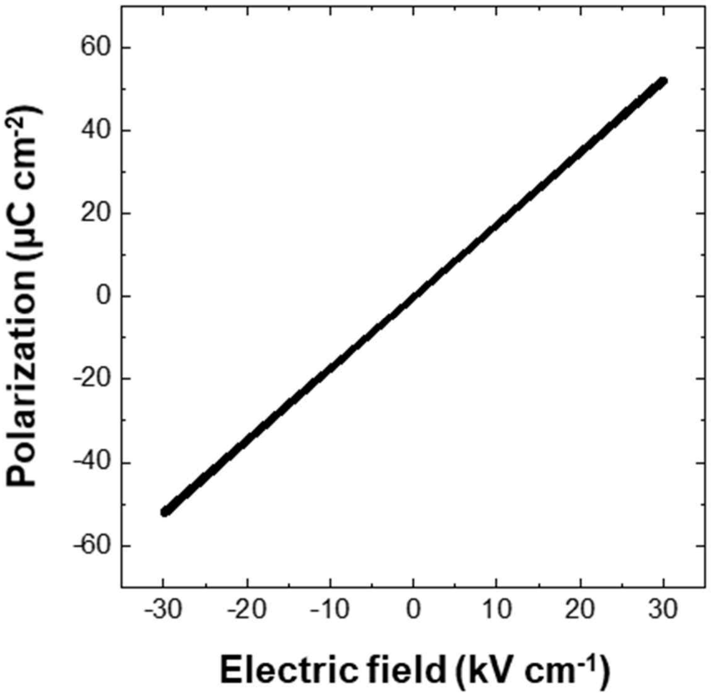

Supplementary Figure 8. In-plane polarization measurement versus electric field. Room temperature hysteresis loop for two parallel electrodes on the bare PSO substrate. The electric field is applied parallel to PSO $[002]_O$. There is a linear relationship between polarization and applied electric field, indicating that the overall positive slope in the BTO/PSO sample shown in Fig. 4b and c is originated from the PSO substrate.

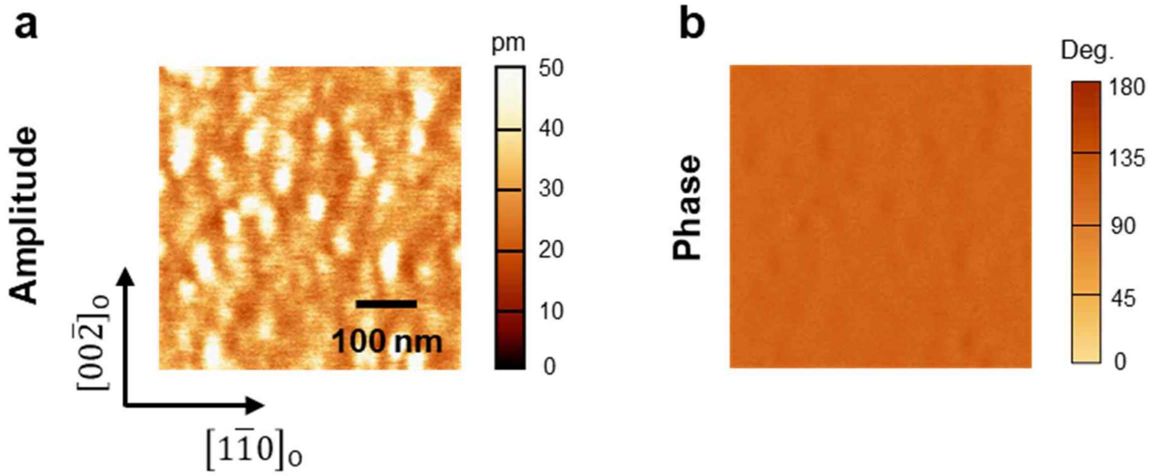

Supplementary Figure 9. Vertical PFM images of the BTO film on PSO substrate. **a,** amplitude image. **b**, phase image.

Supplementary Note 3: Domain structure along the PSO $[002]_O$ and its origin

Angular dependent lateral PFM imaging of the domain structures are shown in Supplementary Figure 10. In the lateral PFM mode, signal is sensitive only to the polarization perpendicular to the cantilever arm, so that the measured domain structure represents a projected polarization along the axis perpendicular to the cantilever arm (polarization projection axis). A physical rotation of the sample relative to the cantilever arm was adopted in Supplementary Figure 10 while imaging in the exact same area, and the polarization directions sensitive to the specific sample-cantilever arm angle are represented by dotted arrows (Supplementary Fig. 10f-j). First, the PFM image taken with polarization projection axis along PSO $[1\bar{1}0]_O$ (referred to as 0° angle) exhibit no contrast, indicating a single domain state (Supplementary Fig. 10f). By rotating the relative sample-cantilever arm angle, the small portion of contrast begins to appear as the angle reaches 75° (Supplementary Fig. 10h). The PFM image taken at 90° (with polarization projection along the PSO $[002]_O$ direction) shows both up and down domain (Supplementary Fig. 10i). The data with 105° represent similar tiny domain feature with to that of 75° (Supplementary Fig. 10j). From this angular dependent PFM imaging results, we conclude that the in-plane polarization direction is mainly toward PSO $[1\bar{1}0]_O$, with small polarization tilting angles toward PSO $[002]_O$ and PSO $[00\bar{2}]_O$ of a magnitude less than 15° in majority of the area. Supplementary Figure 11 shows the reconstructed image of the actual polarization orientation (with the angle relative to PSO $[1\bar{1}0]_O$ axis) from analysis of PFM images, and a histogram distribution of the polarization angles within the inspected area.

This non-zero polarization along PSO $[002]_O$ direction is also supported by high magnification HAADF-STEM Images of a BTO film along the PSO $[1\bar{1}0]_O$ zone-axis (Supplementary Figure 12). Ti displacements toward both PSO $[002]_O$ and $[00\bar{2}]_O$ direction are observed (Supplementary Figure 12c, d). It should be noted that the magnitude of Ti displacement along the PSO $[002]_O$ and $[00\bar{2}]_O$ is much smaller than that of along the PSO $[1\bar{1}0]_O$ (Fig. 3e, f). In addition, the overall in-plane domain structure is consistent with phase field simulation (Supplementary Figure 13b). When BTO is coherently grown on PSO $(110)_O$, *c*-axis (long axis) of bulk tetragonal BTO is along PSO $\langle 1\bar{1}0\rangle_O$ under compressive strained (Supplementary Figure 13a) while *a*/*b*-axis (short axis) of bulk tetragonal BTO is along PSO $\langle 002\rangle_O$ under tensile strained state. This may lead to form zigzag patterns of in-plane domain structure as shown in Supplementary Figure 13b and 14. This explanation is further supported by simulation results performed under absence of tensile strain along the PSO $\langle 002\rangle_O$ direction (Supplementary Figure 13c). The result shows a pure single domain structure where polarization direction is perfectly toward to PSO $[1\bar{1}0]_O$ direction (Supplementary Figure 13d and 15), indicating the validity of our strategy.

It should be noted that the domain pattern tends to become more complex with additional extrinsic factors at play. Specifically, when PFM analysis is performed closer to the edge of the sample, a polydomain structure arises due to the large-scale periodic changes of the polarization direction along PSO $[\bar{1}10]_O$ direction (Supplementary Figure 16). It is feasible to assume that the

elastic strain conditions are quite different at the vicinity (in the range of several hundreds of micrometers) of the substrate edge in comparison to the substrate interior area. In this case, more complex patterns of strain anisotropy and lattice distortion would create conditions for domain periodicity along different crystallographic directions. Detail analysis of the effect of such extrinsic factors on the in-plane polarization alignment are underway.

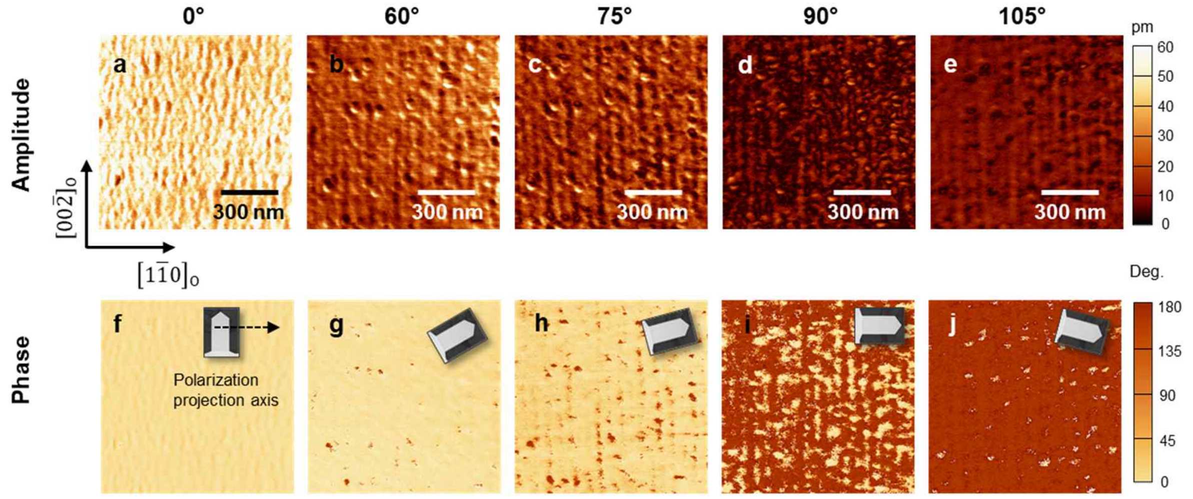

Supplementary Figure 10. Angular dependent LPFM (a-e) amplitude and (f-j) phase images of the BTO film on PSO substrate at the same location. The relative sample-polarization projection axis angles: 0° (used as a reference) (**a, f**) , 60° (**b, g**), 75° (**c, h**), 90° (**d, i**), 105° (**e, j**).

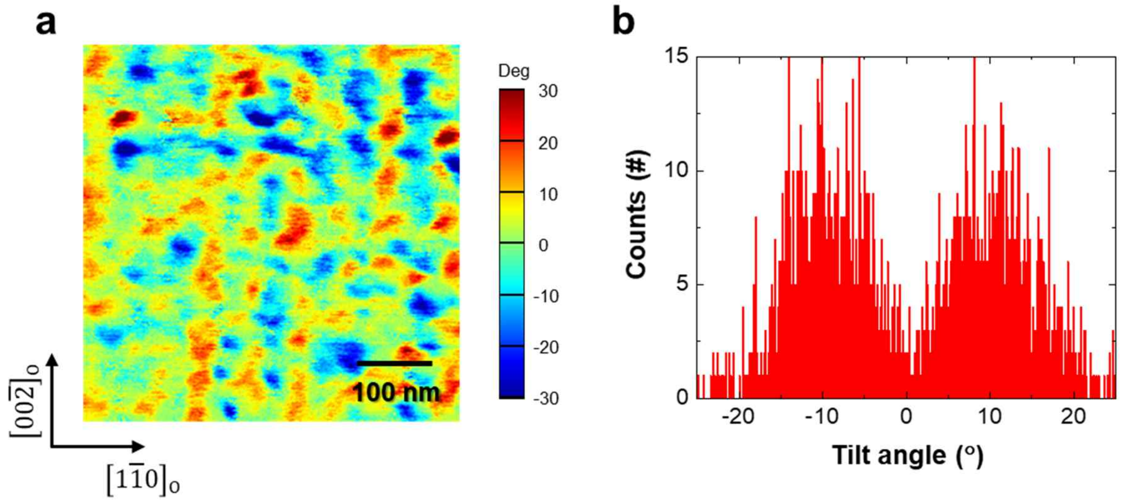

Supplementary Figure 11. Actual polarization angles relative to PSO $[1\bar{1}0]_O$ direction. **a**, Reconstructed polarization orientation map relative to PSO $[1\bar{1}0]_O$ direction from the PFM images. **b**, Histogram distribution of the polarization tilting angle relative to PSO $[1\bar{1}0]_O$ direction.

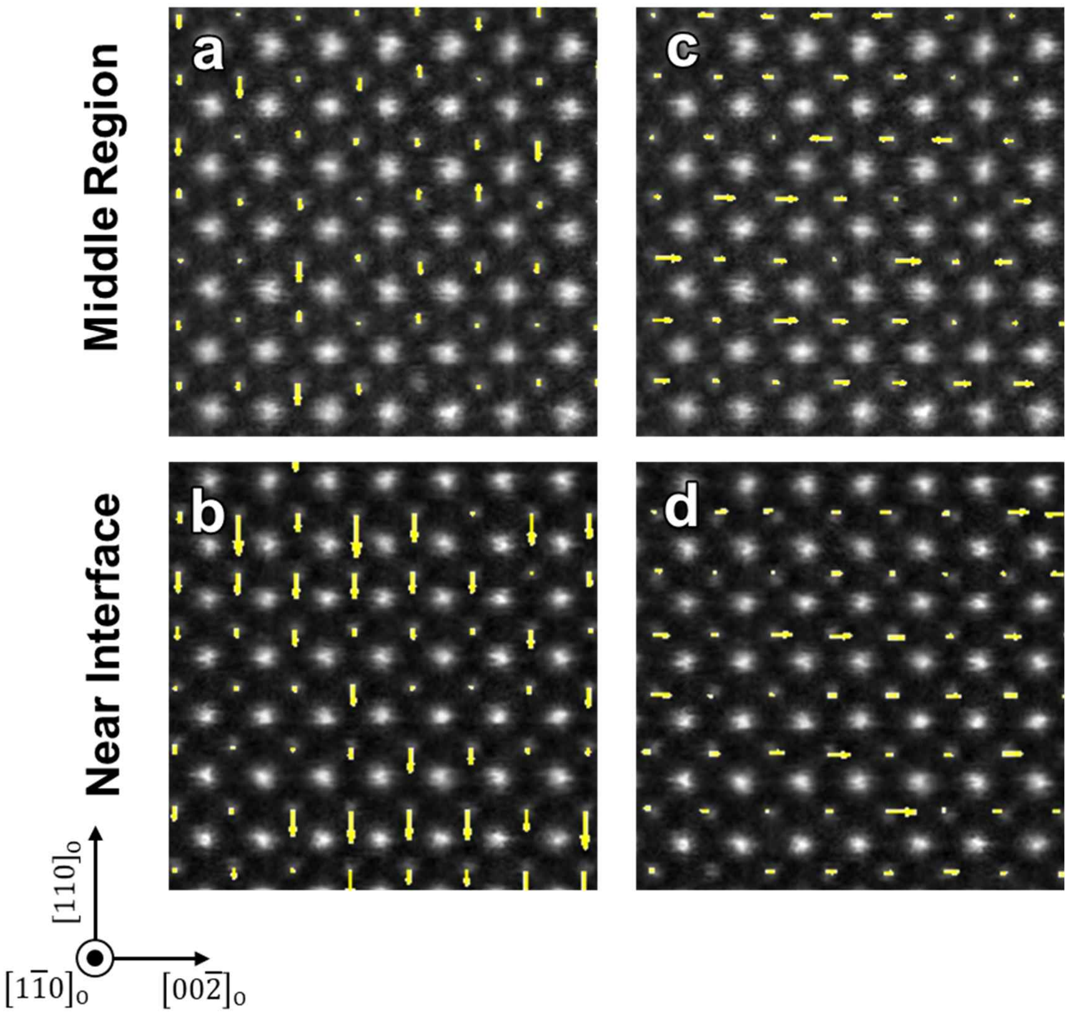

Supplementary Figure 12. HAADF-STEM images of BTO film with a zone axis of PSO $[1\bar{1}0]_O$. **a**, **b**, Out-of-plane component of Ti-displacement in the middle region (**a**) and near interface region (**b**) of the BTO film, respectively. **c**, **d**, In-plane component of Ti-displacement in the middle region (**c**) and near interface region (**d**) of the BTO film, respectively.

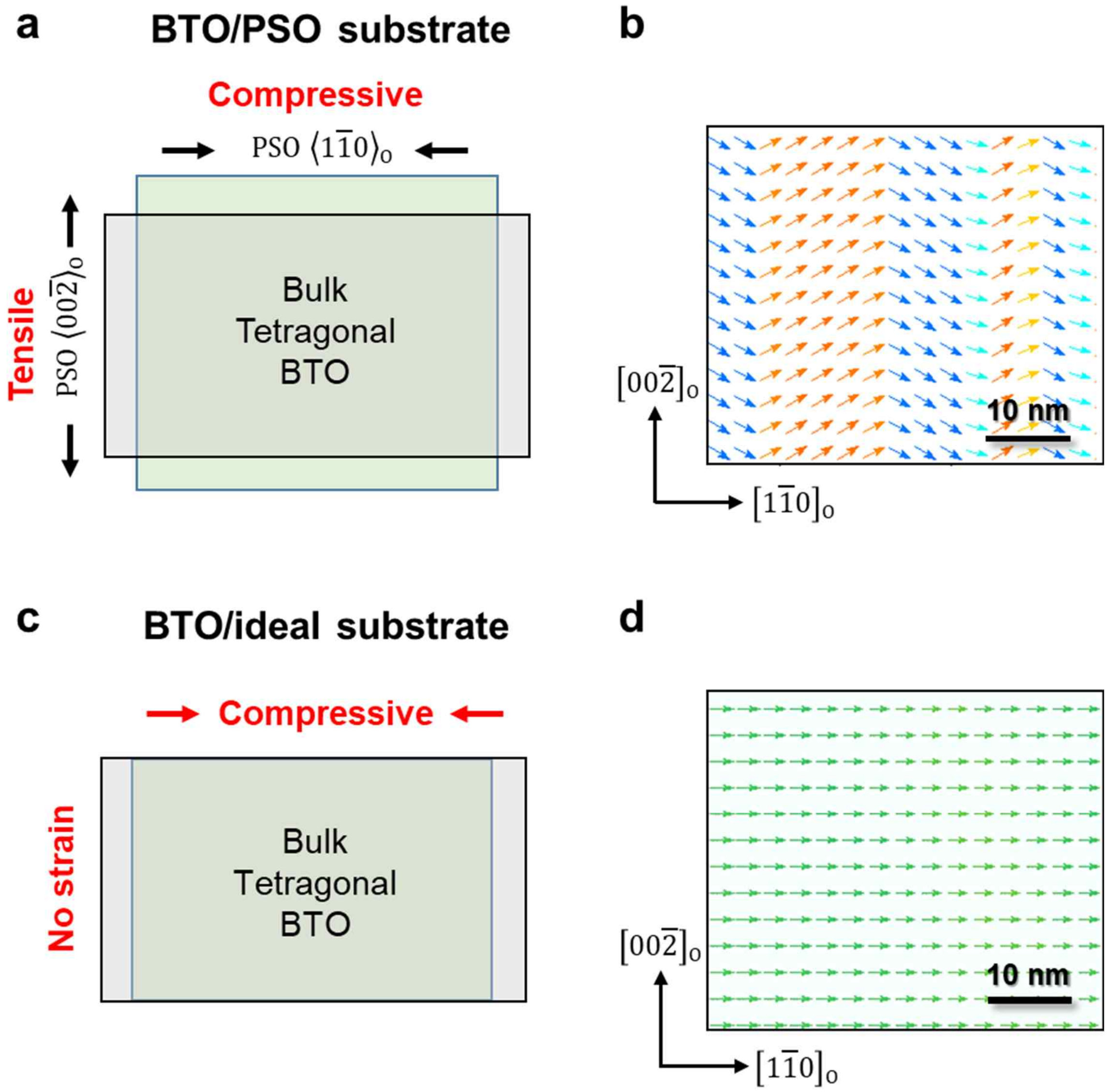

Supplementary Figure 13. In-plane domain structure simulated by phase field simulation. **a**, The schematic of the strain relationship between bulk tetragonal BTO and PSO $(110)_O$ substrate. **b,** Calculated zigzag domain patterns in BTO/PSO $(110)_O$ substrate. **c**, The schematic of BTO grown on the ideal substrate. **d**, Calculated single in-plane ferroelectric domain in BTO/ideal substrate.

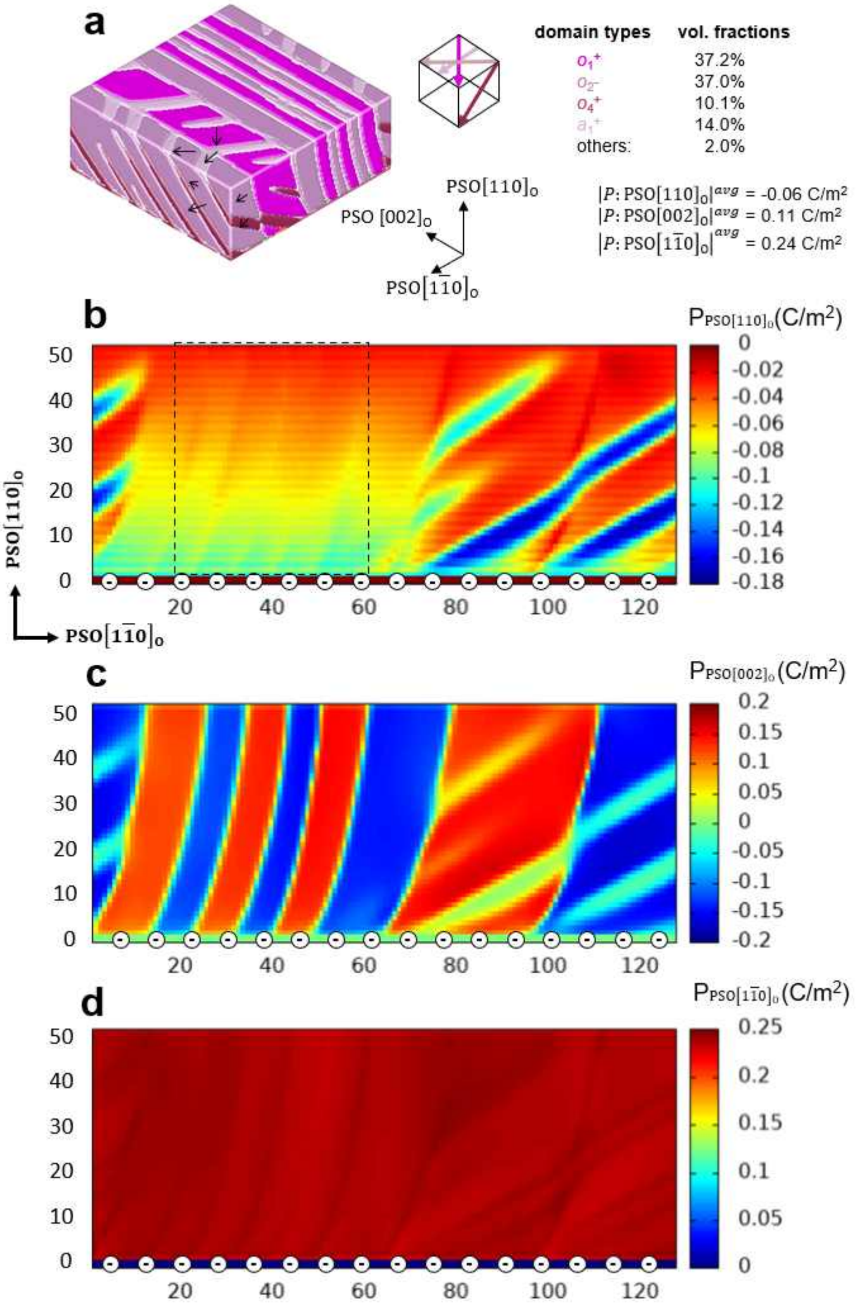

Supplementary Figure 14. Phase-field simulation of the 50 nm BTO thin film on PSO $(110)_O$ substrate. a, The three-dimensional domain structure of the BTO thin film at equilibrium. The colors represent different domain variants as determined by the direction of polarization vectors, as shown in the legend. The volume fraction of each domain variant and the averaged polarization magnitude along the three orthogonal direction of the system are given. **b-d**, The mappings of polarization vectors for **P**:PSO $[110]_O$ (**b**), **P**:PSO $[002]_O$ (**c**), and **P**:PSO $[1\bar{1}0]_O$ (**d**) components across a two-dimensional section in the PSO $[1\bar{1}0]_O - [110]_O$ plane. The negatively charged interfacial layer is schematically drawn in (**b-d**). The dashed rectangular region in (**b**) denotes the selected region shown in Figure 2 of the main text.

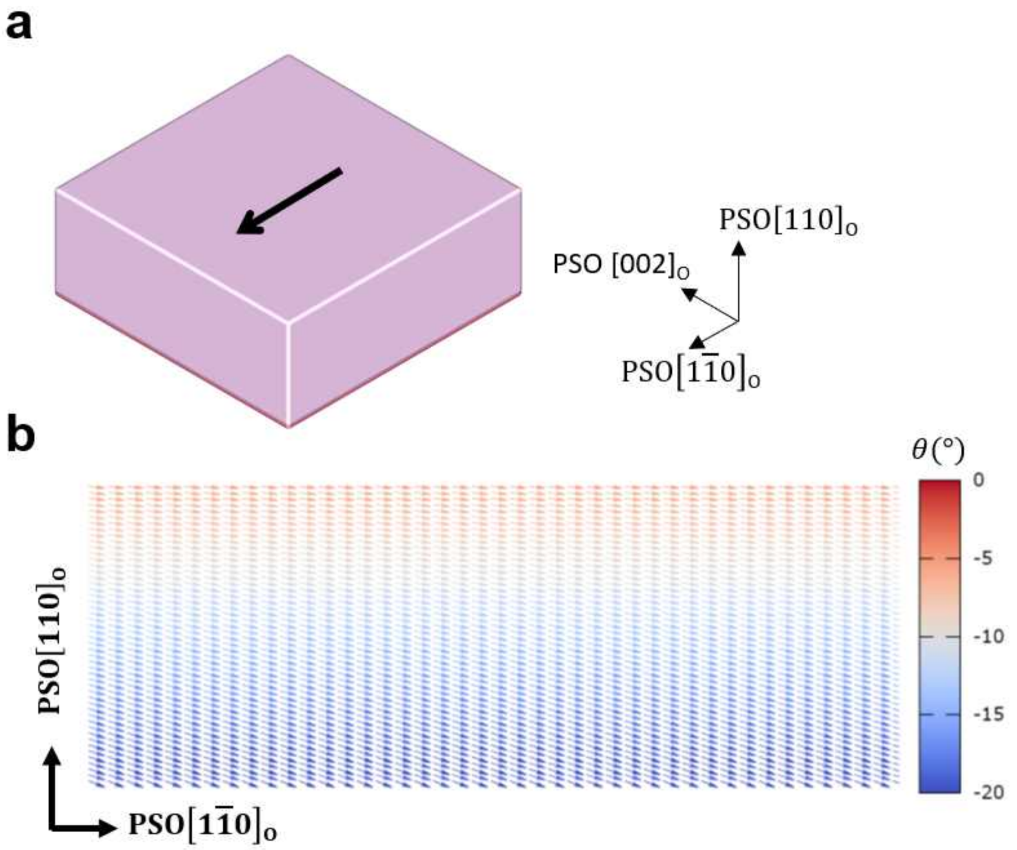

Supplementary Figure 15. Phase-field simulation of the 50 nm BTO thin film on ideal substrate. **a**, The 3D domain structure of the BTO thin film at equilibrium, which shows a uniform a_1^+ domain with $P \parallel$ PSO $[110]_O$. **b**, The section view of the distribution of polarization vectors colored by the rotation angle of polarization with respect to the PSO $[1\bar{1}0]_O$ direction.

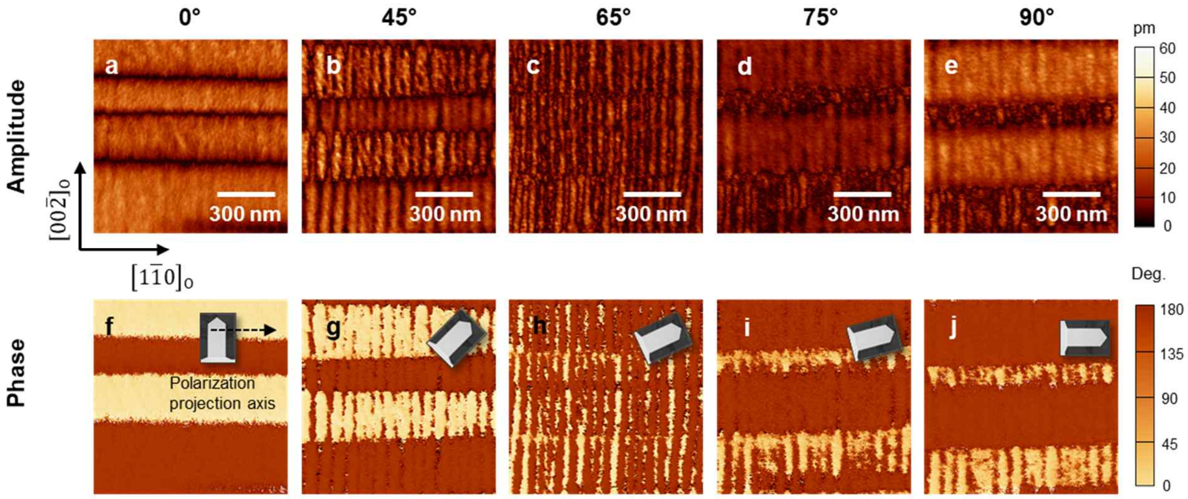

Supplementary Figure 16. Angular dependent LPFM (a-e) amplitude and (f-j) phase images of the BTO film on PSO substrate near the edge region. The relative sample-polarization projection axis angles: 0° (used as a reference) (**a, f**) , 45° (**b, g**), 65° (**c, h**), 75° (**d, i**), 90° (**e, j**).

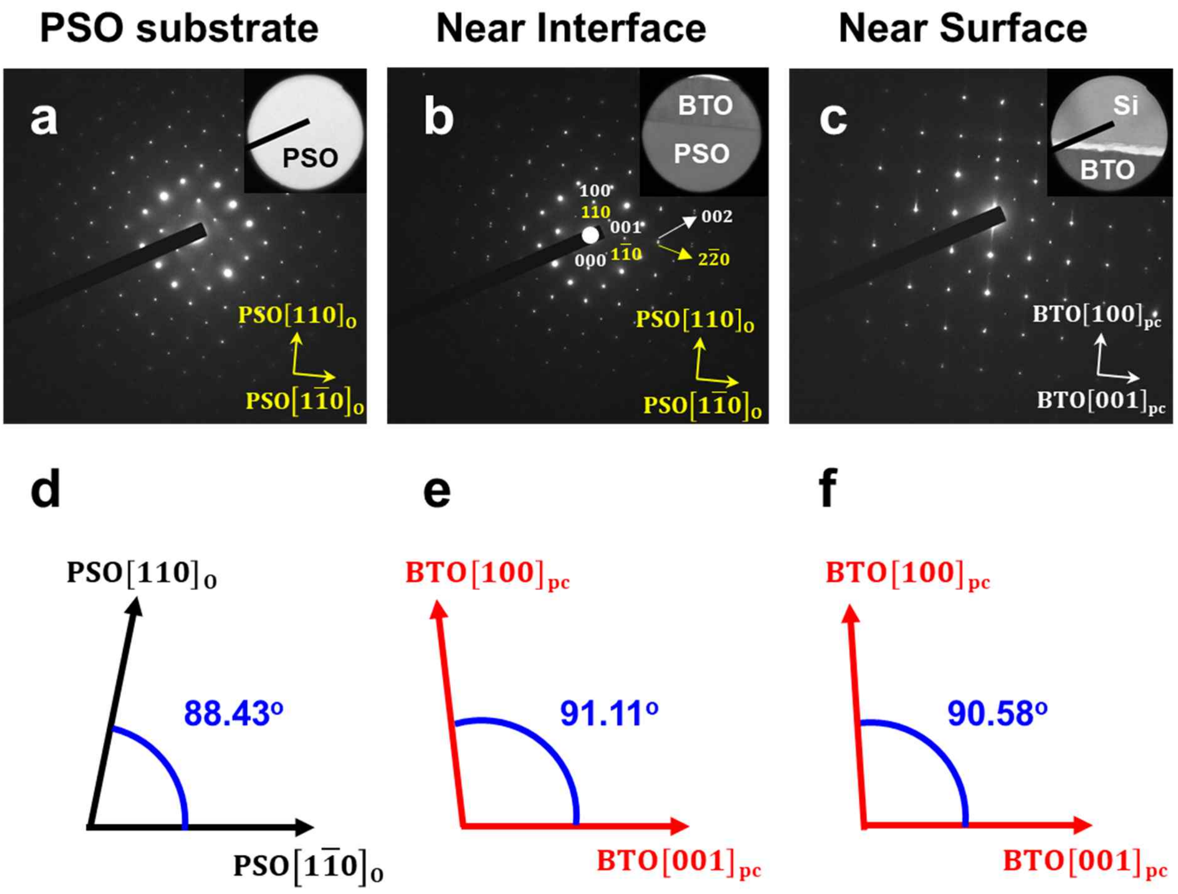

Supplementary Figure 17. The diffraction patterns and tilt angles of PSO and BTO with a zone axis of PSO $[002]_O$. a-c, Diffraction patterns for PSO substrate (**a**), at the interface between BTO/PSO (**b**), and near the top surface of BTO film (**c**). **d–f,** The angle between in-plane and out-of-plane axis of PSO substrate (**d**), BTO near the interface region (**e**), BTO near the surface region (**f**). Note that monoclinic tilting is decreased in BTO near surface region, indicating the structural relaxation.

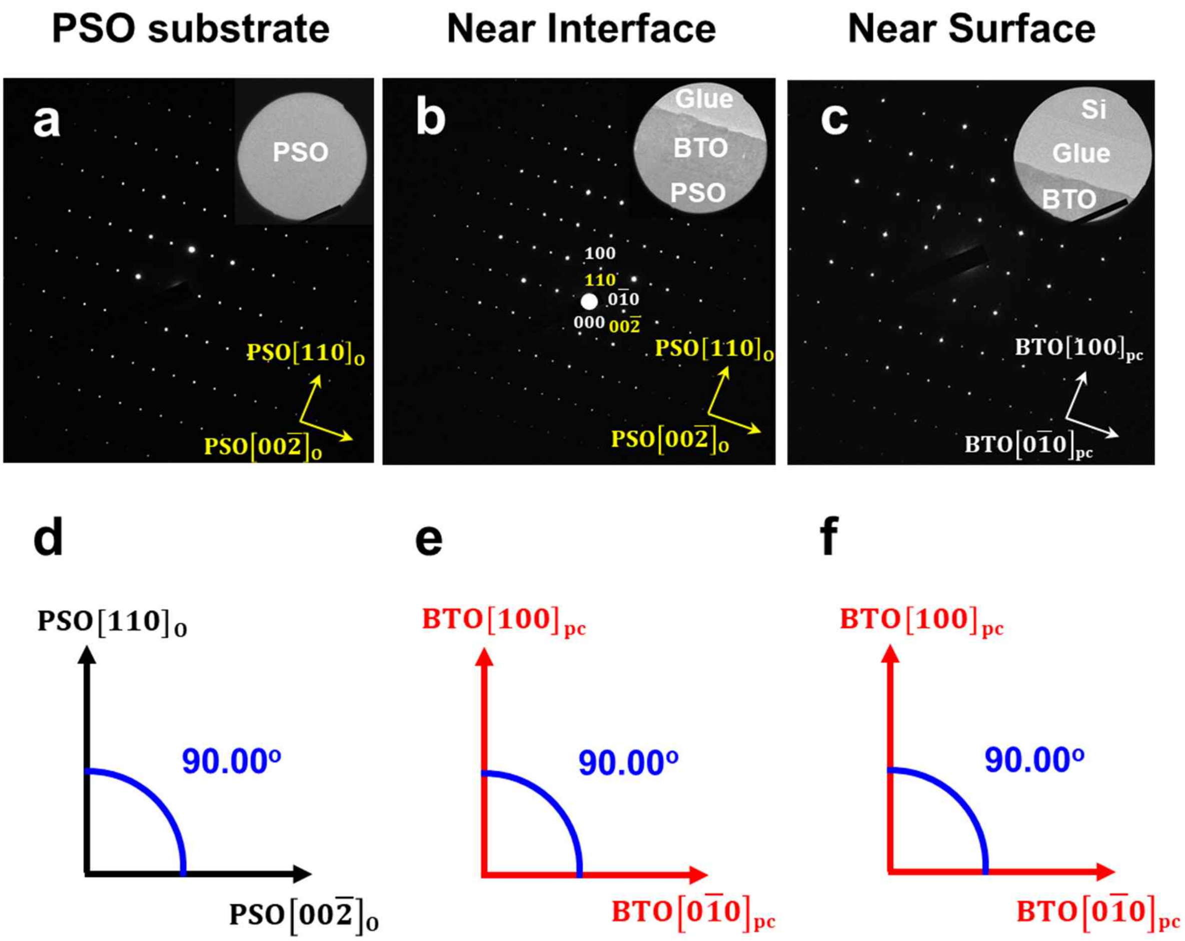

Supplementary Figure 18. The diffraction patterns and tilt angles of PSO and BTO with a zone axis of PSO $[1\bar{1}0]_O$. a-c, Diffraction patterns for PSO substrate (**a**), at the interface between BTO/PSO (**b**) and near the top surface of BTO film (**c**). **d–f**, The angle between in-plane and out-of-plane axis of PSO substrate (**d**), BTO near the interface region (**e**), BTO near the surface region (**f**). Note that there is no monoclinic tilt in BTO both near interface and top surface regions.

Supplementary Note 4: The strain analysis of the BTO film on PSO $(110)_O$ substrate

Based on our model, there is a strain gradient in our BTO film on PSO $(110)_O$ substrate with respect to thickness direction. This strain gradient is also observable in geometric phase analysis (GPA) from TEM data. As shown in Supplementary Figure 19(b), it is clear that BTO film is under higher tensile strain state near interface, which is relaxed within 10 nm (Supplementary Figure 19(d)). This is consistent with our model where electric field across the interface can affect the interface region of BTO causing downward polarization, and then might be weaker in the middle region of the films. On the other hand, there is no strain gradient along in-plane direction (Supplementary Figure 19(c) and (e)), because our BTO film is fully coherent with PSO $(110)_O$ substrate.

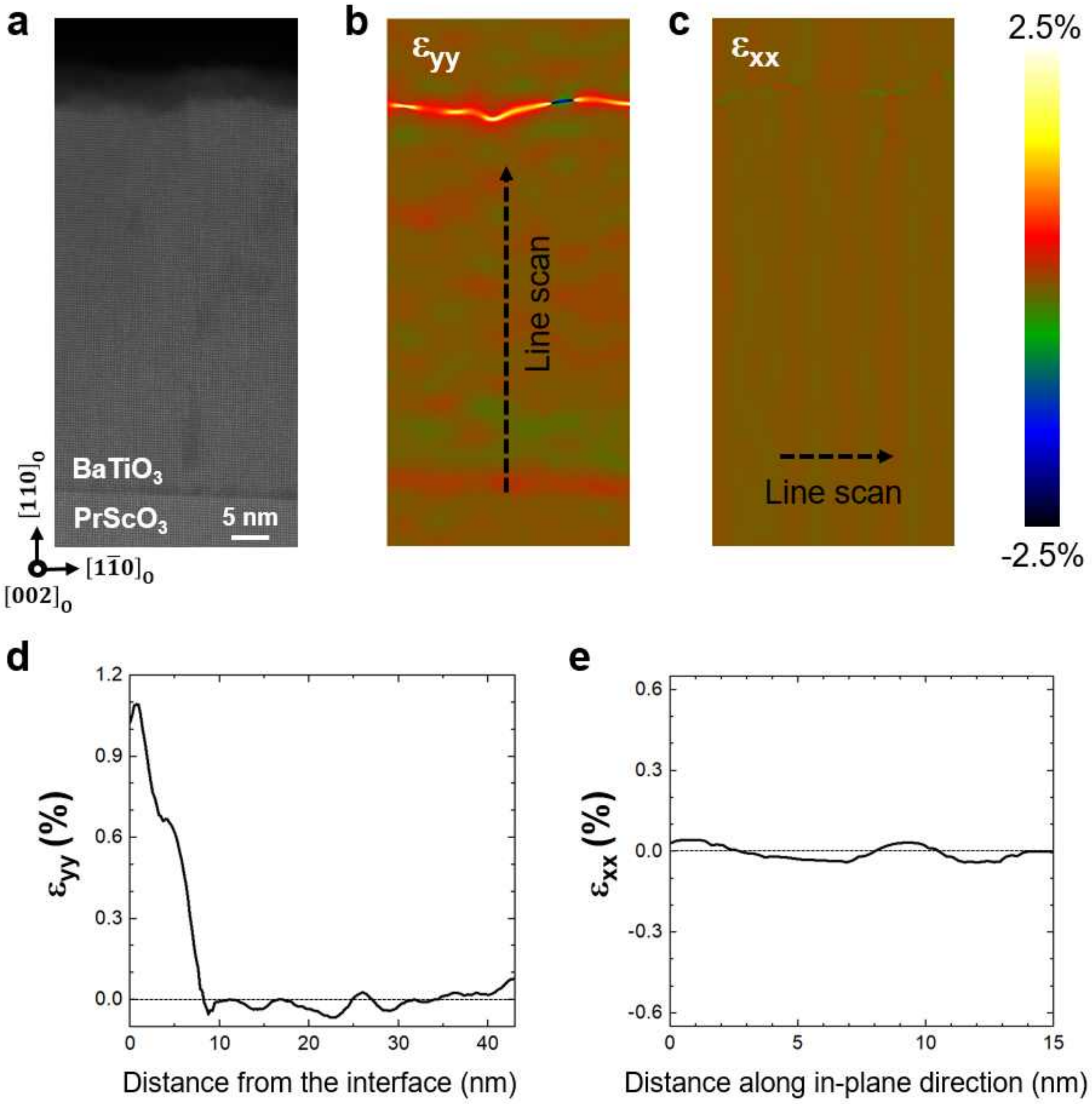

Supplementary Figure 19. Geometric phase analysis (GPA) of the BTO film. a, Low magnification HAADF STEM image of the BTO thin film. **b**,**c**, Corresponding GPA analysis along (**b**) y direction (out-of-plane) and (**c**) x direction (in-plane), respectively. The striped patterns in (**c**) are caused by scanning noise in the STEM image. **d**,**e**, Line scanning result of (**d**) ε_{yy} as a function of distance from the interface, and (**e**) ε_{yy} as a function of distance along in-plane direction.

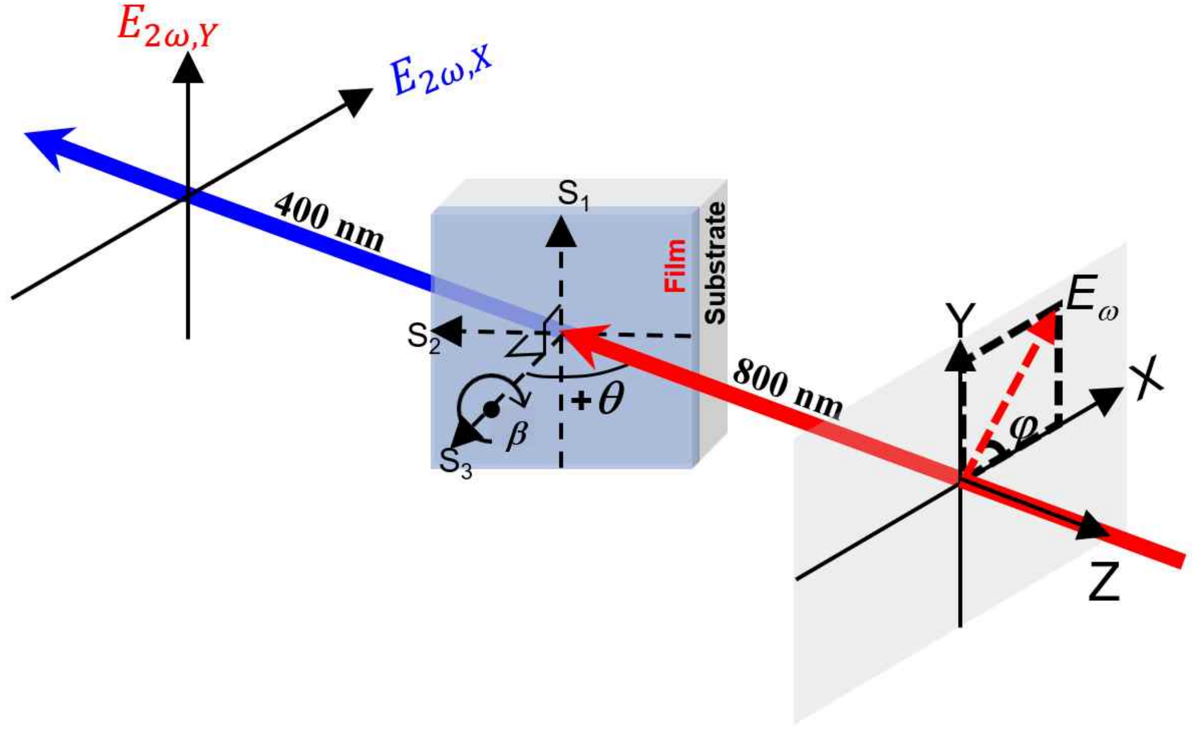

Supplementary Figure 20. Schematic of far-field optical second harmonic generation setup. Linear polarized fundamental optical beam at λ = 800 nm is incident onto the sample at an angle θ. Transmitted X-/Y-polarized SHG signal at λ = 400 nm is measured as rotating the polarizing direction φ.

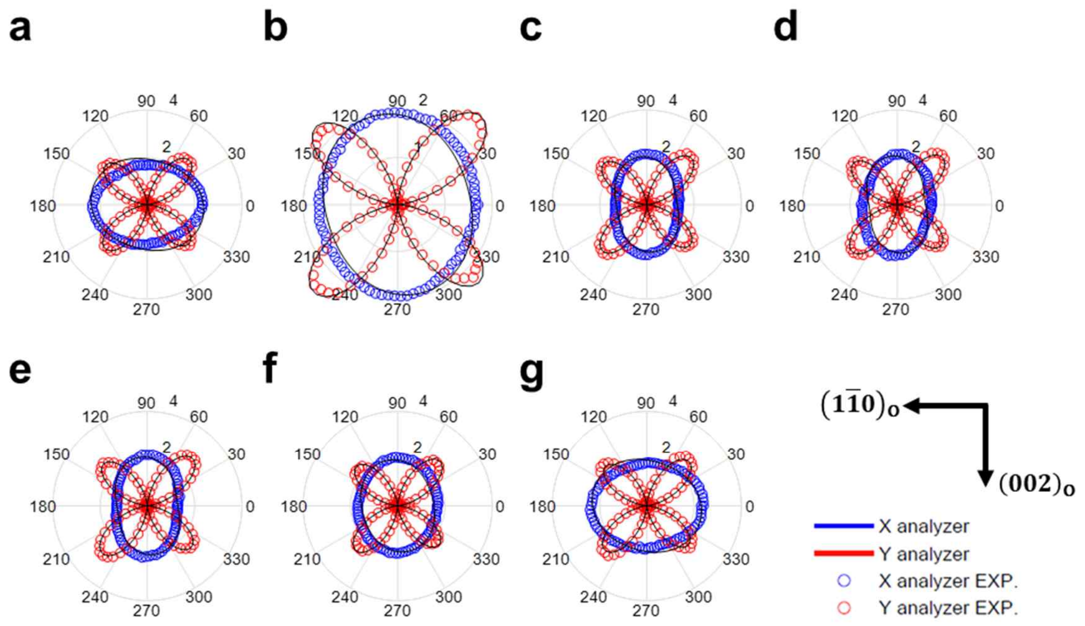

Supplementary Figure 21. SHG polar plots of the BTO film on PSO $(110)_O$ substrate. **a-g**, plots with an incident angle of -45° (**a**), -30° (**b**), -15° (**c**), 0° (**d**), 15° (**e**), 30° (**f**), and 45° (**g**). The solid lines are the fits assuming a monoclinic symmetry for BTO.

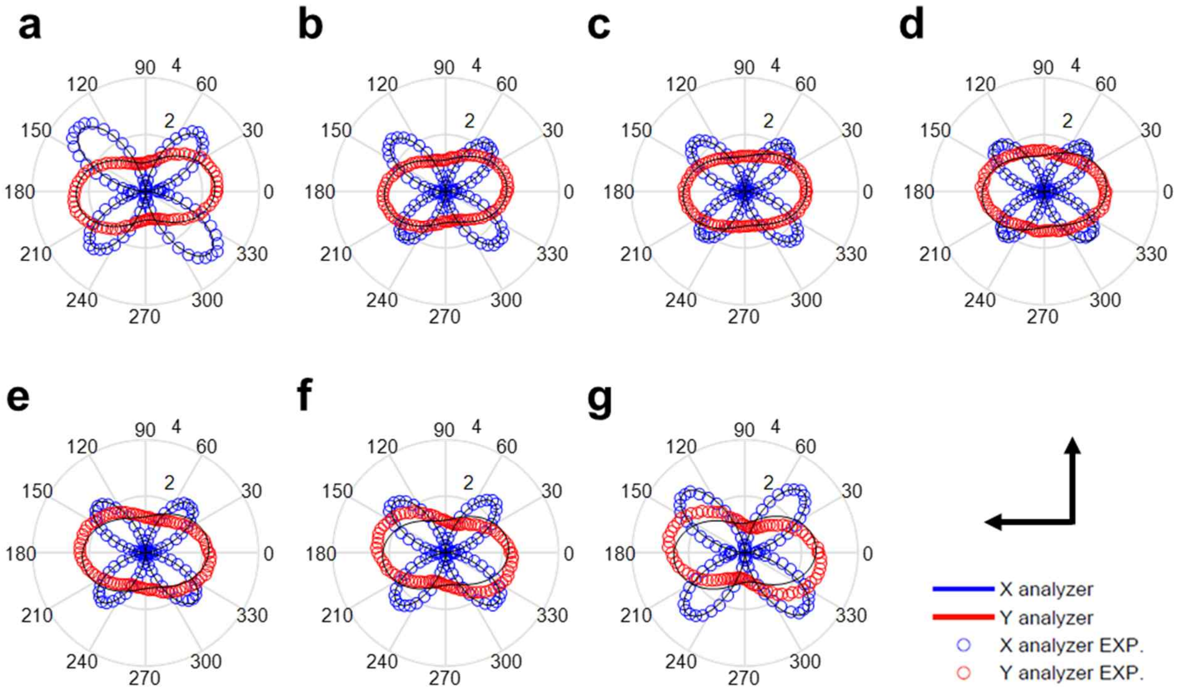

Supplementary Figure 22. SHG polar plots of the BTO film on PSO $(110)_O$ substrate with different in-plane orientation. **a-g**, plots with an incident angle of -45° (**a**), -30° (**b**), -15° (**c**), 0° (**d**), 15° (**e**), 30° (**f**), and 45° (**g**). Note that the in-plane sample orientation is rotated by 90° as compared to the data in Supplementary Figure 21. The solid lines are the fits assuming a monoclinic symmetry for BTO. The good quality of the fits as shown by the black solid lines in Supplementary Figure 21, 22 suggests the BTO film indeed exhibits a single ferroelastic domain with monoclinic distortion, which is consistent with STEM observations.

Supplementary Table 1. Anisotropic misfit strain between $BaTiO_3$ and $REScO_3$ (RE = La, Ce, Pr, Nd or Gd) substrates. The lattice parameters were referred from ref. 6, 7, except PSO. Pseudocubic lattice parameters of PSO were measured by XRD, using the single crystal substrate which was used in our experiment.

Material	a_{pc} (Å)	b_{pc} (Å)	c_{pc} (Å)	c_{pc}/b_{pc}	S_b (%) $(b_{RESO}-b_{BTO})/b_{BTO} \times 100$	S_c (%) $(c_{RESO}-c_{BTO})/c_{BTO} \times 100$
$BaTiO_3{}^T$	3.992	3.992	4.036	1.011		
$LaScO_3{}^O$	4.053	4.049	4.053	1.001	+1.43	+0.42
$CeScO_3{}^O$	4.036	4.023	4.036	1.003	+0.78	0.00
$PrScO_3{}^O$	4.026	4.007	4.026	1.005	+0.38	–0.25
$NdScO_3{}^O$	4.013	4.000	4.013	1.003	+0.20	–0.57
$GdScO_3{}^O$	3.973	3.967	3.973	1.002	–0.63	–1.56

pc: pseudocubic, T: tetragonal, O: orthorhombic

$a_{pc}//[110]_O$, $b_{pc}//[002]_O$, $c_{pc}//[1\bar{1}0]_O$

S_b: misfit strain in *b*-axis of BTO, S_c: misfit strain in *c*-axis of BTO

Supplementary References

1. Liferovich, R. P. & Mitchell, R. H. A structural study of ternary lanthanide orthoscandate perovskites. *J. Solid State Chem.* **177**, 2188–2197 (2004).
2. Glazer, A. M. The Classification of Tilted Octahedra in Perovskites. *Acta Cryst.* **B28**, 3384–3392 (1972).
3. Coh, S. *et al*. Si-compatible candidates for high-κ dielectrics with the P*bnm* perovskite structure. *Phys. Rev. B* **82**, 064101 (2010).
4. Aso, R., Kan, D., Shimakawa, Y., & Kurata, H. Octahedral Tilt Propagation Controlled by A-site Cation Size at Perovskite Oxide Heterointerfaces. *Cryst. Growth Des.* **14**, 2128–2132 (2014).
5. Lee, H. *et al*. Imprint Control of $BaTiO_3$ Thin Films via Chemically Induced Surface Polarization Pinning. *Nano Lett.* **16**, 2400–2406 (2016).
6. Choi, K. *et al*. Enhancement of ferroelectricity in strained $BaTiO_3$ thin films. *Science* **306**, 1005–1009 (2004).
7. Schubert, J. *et al*. Structural and optical properties of epitaxial $BaTiO_3$ thin films grown on $GdScO_3$(110). *Appl. Phys. Lett*. **82**, 3460–3462 (2003).
